# Da Vinci Single-Port robot-assisted transanal mesorectal excision: a promising preclinical experience

**DOI:** 10.1007/s00464-020-07444-4

**Published:** 2020-05-11

**Authors:** Werner Kneist, Hubert Stein, Markus Rheinwald

**Affiliations:** 1 Department of General, Visceral and Transplant Surgery, University Medicine of the Johannes Gutenberg-University, Mainz, Germany; 2 Department of General and Visceral Surgery, St. Georg Hospital Eisenach gGmbH, Eisenach, Germany; 3 Department of Global Clinical Development, Intuitive Surgical, Sunnyvale, CA USA; 4 Department of General and Visceral Surgery, St. Georg Hospital Eisenach gGmbH, Mühlhäuser Straße 94, 99817 Eisenach, Germany

**Keywords:** Robotics, Single-port surgery, Robotic transanal surgery, Rectal cancer, Transanal total mesorectal excision (taTME), Da vinci SP

## Abstract

**Introduction:**

Robotic single-port platforms represent a viable option for advanced surgical procedures. This preclinical study investigated the dual-field, single-port, robot-assisted transanal total mesorectal excision (taTME).

**Technique:**

In a male human cadaver, we employed the novel da Vinci® SP™ Surgical System, sequentially, to realize the transanal and abdominal parts of the taTME procedure. We evaluated the feasibility of the one-team approach.

**Results:**

We showed that single-port access for the taTME was technically feasible with the current da Vinci® SP™ Surgical System in both surgical fields. The total console times were 189 min for the juxta-anal purse-string suture placement, partial intersphincteric resection, and bottom-up mesorectal dissection to where it meets the peritoneal reflection and 43 min for the abdominal procedure. A good quality specimen was achieved. The surgeon comfort was high during simulated surgery. The task load was highly acceptable (NASA-TLX global score: 35), even though it was the surgeon’s first use of this platform.

**Conclusion:**

This preclinical study demonstrated that the robotic, single-port taTME was feasible and could be performed with the da Vinci® SP™ Surgical System, beginning at the level of the dentate line. Further simulations are necessary to confirm this promising approach.

**Electronic supplementary material:**

The online version of this article (10.1007/s00464-020-07444-4) contains supplementary material, which is available to authorized users.

Compared to traditional approaches, dedicated, single-port, robotic platforms incorporate a higher range of movement for cameras, complex articulating instruments, and devices. Thus, they promise greater minimal invasiveness, more precise surgery, and more comfortable surgery in the thoracic, abdominal, and pelvic cavities. The first meta-analysis on clinical studies that employed single- and multi-port robot-assisted platforms revealed that those two approaches resulted in similar conversion and complication rates. Current limits could be superseded by consistently implementing technical innovations and taking the learning curves seriously [1]. Furthermore, different stages of evolution are necessary to evaluate innovative surgical approaches and related complex medical devices. The surgical assessment and the long-term monitoring of the transanal total mesorectal excision (taTME) for individualized, minimally invasive rectal cancer treatment is a good example of this [2–5]. Because new medical technology solutions can increase the potential of surgical innovations, iterative modification loops should be considered. A recent study evaluated matching data from 226 laparoscopic-assisted taTMEs with 370 multi-port robotic TMEs from five high-volume rectal cancer specialist institutions. They showed that these two methods were equivalent in the achievement of high-quality TMEs. Noteworthy, a small taTME subgroup with patients underwent either abdominal (*n* = 10) or transanal robotic assistance (*n* = 5) was included [6]. Additionally, totally robotic taTME has already been successfully carried out in a pioneering series (*n* = 37) utilizing a second-generation platform (da Vinci Si Surgical System) [7]. However, in the author’s own practice, the abdominal robotic approach with the fourth-generation platform (da Vinci Xi Surgical System) is sometimes combined with the video endoscopic-assisted transanal approach [8].

Against this background, we aimed to investigate the technical feasibility of a single-port robotic platform in a two-field taTME setting with the novel da Vinci® SP™ Surgical System, in a cadaveric model. A video is included to illustrate this preclinical study.

## Materials and methods

For this cadaver study, we carried out a multi-professional, one-day workshop at a certified lab institution with a fully implemented management system for research and development services, both for medical technology industries and advancement and further training of medical and nursing staff (DIN EN ISO 9001:2015). This study complied with the ethical standards and regulations for the use of human body donors, established by the host institution. IRB review was not required for this study as it did not involve human subjects.

The surgeon was familiar with the da Vinci Xi Surgical System (Intuitive Surgical, Sunnyvale, CA, USA), the taTME procedure, and cadaveric simulation. The technical briefing included 30 min of dry lab training for endoscope and instrument navigation procedures with the da Vinci® SP™ Surgical System (Intuitive Surgical, Sunnyvale, CA, USA) and to provide familiarization with the proprietary *SP* holographic navigation aid.

**Fig. 1:**
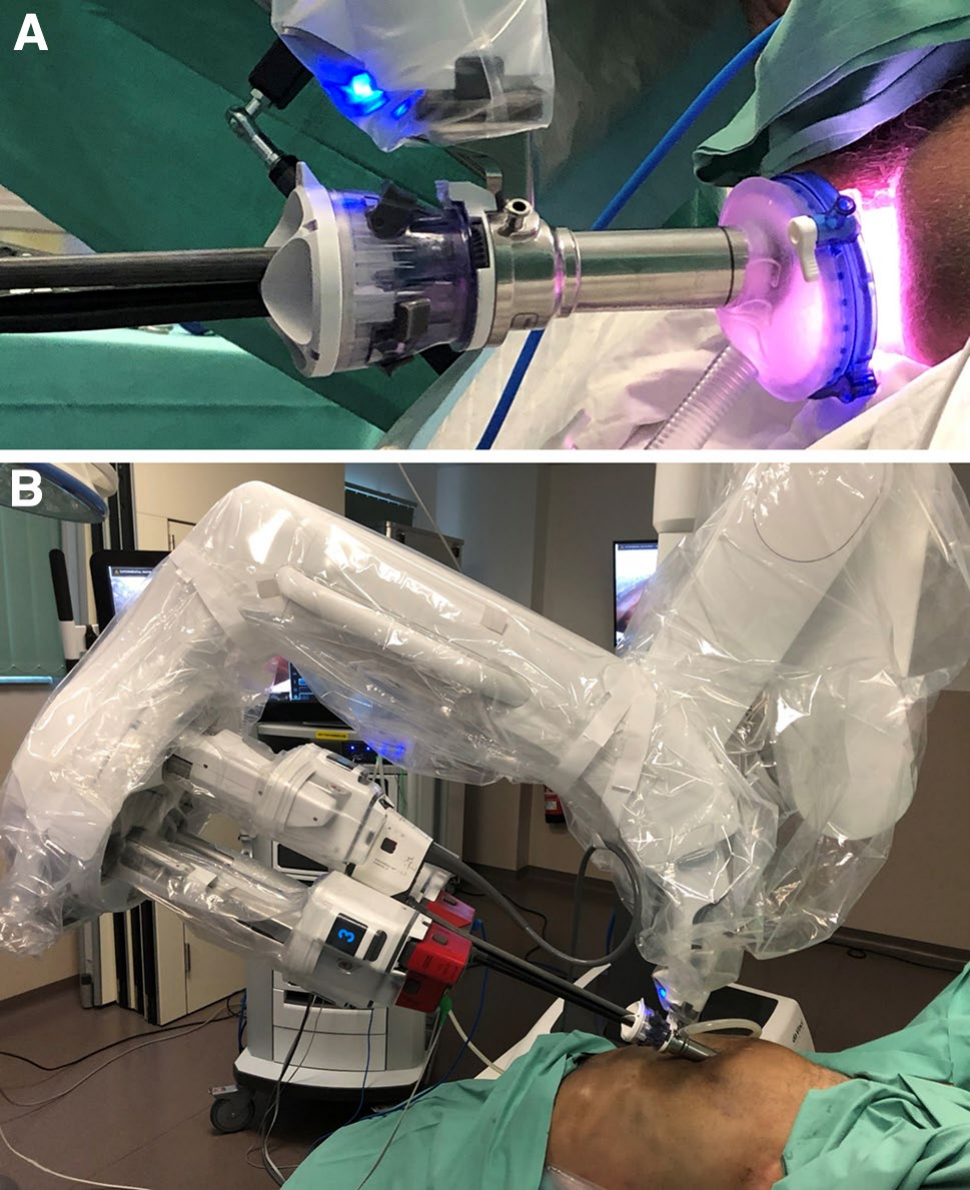
**A** Setup for transanal total mesorectal excision with the GelPoint Path transanal access platform and the da Vinci® SP™ Surgical System; **B** abdominal approach with the single 2.5-cm cannula

After rectal washout, a fresh cadaver (male, 64 years, height 187 cm, weight 59 kg) was placed in the appropriate Lloyd-Davies Position on a mobile operating table (TS 7000dv, Trumpf Medizin Systeme GmbH & Co. KG, Saalfeld, Germany). The taTME was performed with the da Vinci® SP™ Surgical System port placed through the GelPoint Path transanal access platform (channel size 40 × 55 mm; Applied Medical, Rancho Santa Margarita, CA, USA). Briefly, a 12-mm trocar was inserted through the Gelport (Fig. 1). CO_2_ was continuously insufflated to maintain a pressure of 12-mmHg. The robotic abdominal part of the procedure was realized sequentially (one-team approach), whereby the SP port was placed supraumbilical. A separate 12-mm trocar was inserted in the lower right abdominal quadrant. After the specimen was extracted, the TME quality was graded. The workload that was required to perform the totally robotic taTME, single-port procedure in the cadaver model was evaluated by the console surgeon with the NASA-TLX questionnaire [9].

## Results

**Fig. 2:**
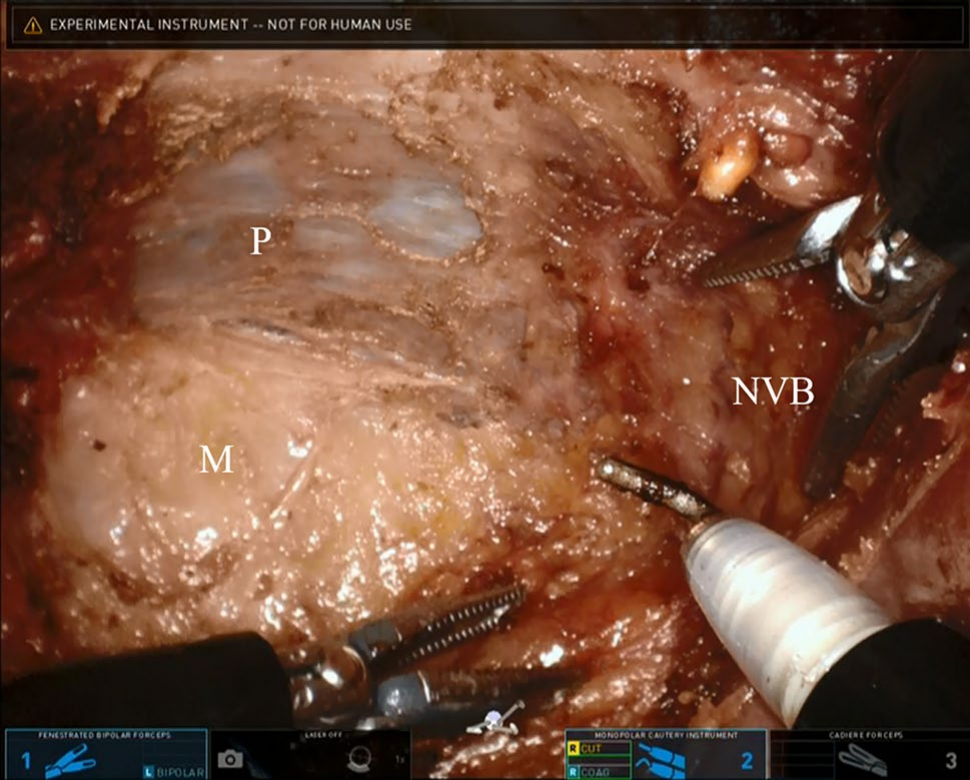
Hook and two fenestrated graspers for transanal anterolateral mesorectal dissection with the da Vinci® SP™ Surgical System. *P* prostate gland, *M* mesorectum, *NVB* neurovascular bundle

The dry run of the SP robotic system and the transanal docking of the left-sided robotic cart was completed without problems. The robot-assisted purse-string suture was placed with two wristed needle drivers to occlude the rectum 1 cm above the dentate line. The knot was tied with a knot pusher, via the 12-mm trocar. Then, the mucosa was incised circumferentially, at the level of the dentate line, 3-cm from the anal verge. This incision and the subsequent partial intersphincteric resection (pISR) were performed with a monopolar hook and a fenestrated grasper, via the SP platform. Then, the transanal mesorectal dissection was achieved with three multi-jointed wristed instruments. (Fig. 2). A subperitoneal circumferential holy plane dissection was completed, without complications, at the level of the *excavatio rectovesicalis*, ventrally, and at the S3 level, dorsally. The transanal part of the operation took 189 min. We recorded one disruption that was required to perform a initial repositioning of the single-port trajectory through the gel pad of the transanal Gelport.

A subsequent abdominal docking was performed without problems. The robotic cart remained on the left, and the second single port was inserted via a 3.5-cm supraumbilical incision, with the instrument arm positioned at the craniolateral right. The robot-assisted abdominal part of the operation was realized with monopolar scissors, two Cadiere bipolar forceps, and a medium large clip applier. This procedure included the mobilization of the sigmoid mesocolon and mesorectal pedicle, down to a dorsal point, where it met the pelvic cavity (S3); a division of Toldt's line; a high-tie ligation of the inferior mesenteric artery; and divisions of the mesenteric vein and mesocolon. The peritoneal fold was opened circumferentially and a mesorectal dissection was completed at the level of the midrectum. The additional 12-mm port was used to transect the left colon with a laparoscopic linear stapler. The TME specimen was in good quality (MERCURY I°). The operative time for the abdominal procedure was approximately 43 min. Table 1 shows the task load rated by the console surgeon.

**Table 1:** NASA Task Load Index, completed by the surgeon to evaluate the work load for a transanal total mesorectal excision, performed with the *da Vinci® SP™ Surgical System* in a male cadaveric model

Scales	Rating	Tally	Weight
Global score	35		
Mental demand	80	4	0.266
Physical demand	20	1	0.066
Temporal demand	40	0	0
Performance	15	5	0.333
Effort	40	2	0.133
Frustration	10	3	0.200

## Discussion

The novel da Vinci® SP™ Surgical System previously received clearance from the Food and Drug Administration for urologic and transoral otolaryngology surgeries. Hence, performing complex gastrointestinal procedures has been limited to the preclinical setting in cadaveric models. Initial results for a transcervical esophagectomy and a transanal submucosal excision, full thickness excision, or cylindrical local excision were promising, particularly for the demanding taTME [10, 11].

In this study, the sticking point was the common indication of a juxta-anal rectal cancer [8], together with inadequate maneuverability in close proximity to the transanal platform. The latter was noted in a previous human cadaver study, where a robotic purse-string suture was realized at 4–5 cm from the anal verge in two cases [12].

In our experiment the articulation of the SP instruments immediate at the juxta-anal area at < 1 cm from the anal ring were initially limited despite the use of an additional transanal access channel to create distance for the instruments to fully unfold for complete range of motion. Nevertheless, the very low purse-string suture and the pISR were feasible robotically with straight configuration of the instruments. This result was consistent with the work of Marks et al. who used a very similar experimental set-up and reported successful local excisions and sutures performed at 1 cm above the dentate line [10]. However, it should be possible to improve the articulation and reduce the anatomically induced risk of dislocation, with individualized adaptations of the single-port and/or the transanal spacer. A specific docking port is currently under development to overcome these issues.

It is important to note that the following mesorectal bottom-up dissection allowed the use of a second grasper to perfect the traction and counter-traction. Currently, the holographic navigator, three flexible robotic arms, and the cobra-like camera are well-recognized benefits of the *SP* platform [10, 11]. Here, we confirmed those benefits in the field of confined subperitoneal surgery. Anterior peritoneal entry was achieved without abdominal assistance. Sequential docking facilitated the single-port, robot-assisted, abdominal colon mobilization and the mesorectal pedicle ligation. The excised specimen showed the complete removal of the mesorectum. In this cadaveric study, the additional 12-mm port was only necessary for knot-pushing and intracorporal stapling. Despite the first-time contact with this next generation platform, a complex two-field surgery with a sequential one-team approach was completed within 4 h. The NASA-TLX cognitive workload assessment by the surgeon resulted in a very acceptable global score of 35. That score was in the range expected (31.9 ± 3.4) for simulated robotic surgery [13]. Further preclinical developments in the field could reduce the surgeon workload, stress, and fatigue associated with sequentially or synchronously performed dual-field robotic surgeries [14].

In summary, this technical note demonstrated the feasibility of a taTME with the da Vinci® SP™ Surgical System in a male cadaveric model. Further preclinical studies are needed to confirm our findings.

## Electronic supplementary material

Below is the link to the electronic supplementary material.

Supplementary file1 (MP4 212623 kb). Video 1 Performance of a transanal total mesorectal excision in a male cadaveric model with the da Vinci® SP™ Surgical System
